# Nanodroplet vaporization with pulsed-laser excitation repeatedly amplifies photoacoustic signals at low vaporization thresholds†

**DOI:** 10.1039/d3ra05639b

**Published:** 2023-12-01

**Authors:** Maria Inês P. Mendes, Carlos D. F. Coelho, Fábio A. Schaberle, Maria João Moreno, Mário J. F. Calvete, Luis G. Arnaut

**Affiliations:** a CQC-IMS, Chemistry Department, University of Coimbra 3004-535 Coimbra Portugal lgarnaut@ci.uc.pt; b LaserLeap Technologies Rua Coronel Júlio Veiga Simão, Edifício B, CTCV, S/N 3025-307 Coimbra Portugal

## Abstract

Nanodroplets' explosive vaporization triggered by absorption of laser pulses produces very large volume changes. These volume changes are two orders of magnitude higher than those of thermoelastic expansion generated by equivalent laser pulses, and should generate correspondingly higher photoacoustic waves (PAW). The generation of intense PAWs is desirable in photoacoustic tomography (PAT) to increase sensitivity. The biocompatibility and simplicity of nanodroplets obtained by sonication of perfluoropentane (PFP) in an aqueous solution of bovine serum albumin (BSA) containing a dye make them particularly appealing for use as contrast agents in clinical applications of PAT. Their usefulness depends on stability and reproducible vaporization of nanodroplets (liquid PFP inside) to microbubbles (gaseous PFP inside), and reversible condensation to nanodroplets. This work incorporates porphyrins with fluorinated chains and BSA labelled with fluorescent probes in PFP nanodroplets to investigate the structure and properties of such nanodroplets. Droplets prepared with average diameters in the 400–1000 nm range vaporize when exposed to nanosecond laser pulses with fluences above 3 mJ cm^−2^ and resist coalescence. The fluorinated chains are likely responsible for the low vaporization threshold, ∼2.5 mJ cm^−2^, which was obtained from the laser fluence dependence of the photoacoustic wave amplitudes. Only *ca.* 10% of the droplets incorporate fluorinated porphyrins. Nevertheless, PAWs generated with nanodroplets are ten times higher than those generated by aqueous BSA solutions containing an equivalent amount of porphyrin. Remarkably, successive laser pulses result in similar amplification, indicating that the microbubbles revert back to nanodroplets at a rate faster than the laser repetition rate (10 Hz). PFP nanodroplets are promising contrast agents for PAT and their performance increases with properly designed dyes.

## Abstract 2

Explosive vaporization of a nanodroplet increases generates a gigantic pressure wave.

## Graphical abstract

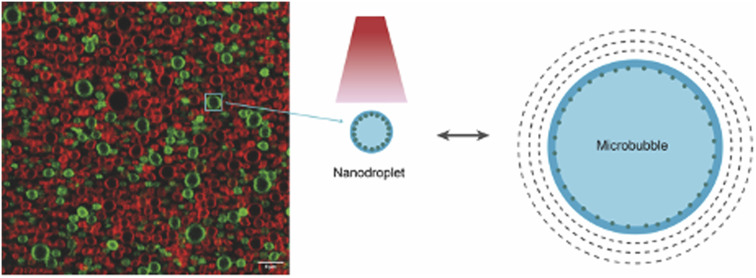

## Introduction

The extraordinary advances in photoacoustic tomography (PAT) over the last decade^1–4^ paved the way for the FDA approval of the first photoacoustic imaging system in 2021.^5^ Initially approved for breast cancer diagnosis, photoacoustic imaging may soon find clinical applications in dermatology and vascular imaging of superficial organs. However, its widespread use as a diagnostic tool is limited by the penetration of light in biological tissues, which restricts photoacoustic imaging depth to <5 cm. Photoacoustic signals are generated when short laser pulses are absorbed by chromophores that rapidly convert optical energy in thermal energy, and the heat released launches a thermoelastic expansion. When the chromophores are embedded in biological tissues and confinement criteria are met,^6^ the thermoelastic expansion generates a photoacoustic wave (PAW) that can be detected by ultrasonic transducers. The ultrasound signals provide information that allows for structural and functional imaging of tissues. PAT takes advantage of electronic absorption to increase contrast and of ultrasonic detection to increase imaging depth.

The thermoelastic expansion of biological structures where endogenous chromophores (*e.g.*, oxy- and deoxyhemoglobin, melanin) absorb nanosecond laser pulses is currently the basis of PAT. This label-free approach is useful for screening large populations but does not exploit the full potential of photoacoustic imaging. Molecular^7,8^ and nanostructured^9^ photoacoustic contrast agents follow the same principles of thermoelastic expansion and can add sensitivity and specificity to PAT. Alternatively, processes such as material ablation and explosive vaporization, which can produce much larger volume changes than thermoelastic expansion, are intrinsically more efficient to generate PAWs,^10^ but they require higher laser fluences and may damage the tissues. In order to place the photoacoustic conversion efficiencies of these processes in perspective, it is useful to express the contributions of thermoelastic expansion^11^ and structural volume change together in the generation of a photoacoustic signal.1
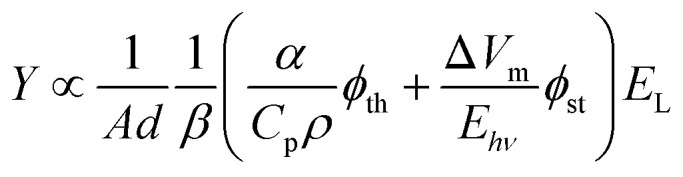
where *A* is the area illuminated, *d* the optical path, *β* the isothermal compressibility (usually in units of cm^2^ dyn^−1^), *α* the thermal expansion coefficient (K^−1^), *C*_p_ the specific heat (J g^−1^ K^−1^), *ρ* the density (g cm^−3^), *E*_L_ the energy of one laser pulse (J), *ϕ*_th_ the efficiency of conversion of optical energy into thermal energy, *ϕ*_st_ the efficiency of generating a structural volume change and *E*_*hv*_ = *hc*/*λ* is the energy of one photon in the laser beam. Using the data for diethyl ether (*α* = 2.15 × 10^−3^ K^−1^, *C*_p_ = 0.556 cal g^−1^ K^−1^, *ρ* = 0.715 g cm^−3^, *β* = 1.38 × 10^−10^ cm^2^ dyn^−1^) and *E*_L_ = 0.5 mJ per pulse, we calculate the change in volume associated with the thermoelastic expansion in eqn (1), as Δ*V*_th_ = 6.5 × 10^−7^ cm^3^. Taking the molar volume change in the photodissociation of diphenylcyclopropenone upon excitation at 355 nm, Δ*V*_m_ ≈ 20 cm^3^ mol^−1^,^12^ as a typical molecular volume change, with the same *E*_L_ we estimate a structural volume change Δ*V*_st_ = 3.5 × 10^−8^ cm^3^. In both cases, *ϕ*_th_ and *ϕ*_st_ were assumed to be unity although it is easier to find radiationless processes with *ϕ*_th_ ≈ 1 than photodissociation reactions with *ϕ*_st_ ≈ 1. This example shows that the structural volume changes of photodissociation reactions are not expected to increase appreciably the intensities of PAWs. However, explosive vaporizations lead to a very different scenario.

Structural volume changes accompanying explosive vaporizations are expected to be very large at ambient pressures because the volume of the liquid is negligible when compared with the volume of the gas. Emelianov and co-workers provided the first evidence that perfluorocarbon (liquid) nanodroplets incorporating a chromophore and coated with bovine serum albumin (BSA), could be vaporized in aqueous solution to (gaseous) microbubbles using laser pulses with fluences currently used in PAT.^13,14^ The liquid-to-gas phase transition increased the photoacoustic signal by more than one order of magnitude relative to that of thermoelastic expansion. Perfluorocarbon droplets stabilized in aqueous media by a shell of surfactants and incorporating organic dyes, metallic nanoparticles, carbon black, graphene oxide or polypyrrole polymers gave similar results.^15–23^ These systems are more suitable to photoacoustic imaging at greater depths than traditional contrast agents. Some authors reported that the first laser pulse may vaporize most of the nanodroplets in the illuminated volume and generate microbubbles, leading to a photoacoustic wave almost one order of magnitude higher than subsequent photoacoustic waves produced exclusively by thermal expansion.^14,15,21^ This is system-specific because other authors reported a fast condensation of the microbubbles back to nanodroplets after the vaporization of nanodroplets to microbubbles.^24–26^ leading to repeatable vaporizations that launch photoacoustic waves consistently larger than those due to thermoelastic expansion. The dynamics of recondensation depends on temperature, droplet size and shell composition, concentration and likelihood of neighboring bubble coalescence. In particular, perfluoropentane (PFP) droplets stabilized with a BSA shell were seen to re-condensate after vaporization when the diameter of the droplet was in the 1–3 μm range but larger droplets showed instability.^25^ Interestingly, the vaporization of droplets with similar composition but an average diameter of 600 nm was not reversible.^15^

Several methods to prepare phase-change contrast agents loaded with absorbing molecules or nanoparticles have been published. Most often, PFP is employed to make perflurocarbon droplets because its boiling point (29 °C) is close to body temperature, its surface tension is low (9.5 mN m^−1^)^27^ and its biocompatibility is excellent. BSA is a frequent choice to stabilize PFP droplets in aqueous solution because albumin works as surfactant and is readily transferred to *in vivo* studies.

This work builds on the knowledge that (i) PFP droplets stabilized by BSA and decorated with dyes can undergo repeated vaporization upon laser pulse excitation, (ii) surfactants with perfluorinated chains contribute to lower interfacial tension between water and PFP droplets more than BSA alone and may lower the vaporization threshold,^28^ (iii) porphyrins bind strongly to BSA without changing the conformation of the protein even after repeated laser pulse excitation,^29^ (iv) free-base porphyrins with long (C_8_F_17_) fluorinated chains were reported to be more soluble in perfluorohexanes^30^ than other dyes (*e.g.*, coumarins, chromene, rhodamine, squaraine) with similar fluorinated chains,^31^ and (v) the complexation of free-base porphyrins with paramagnetic metal ions changes the chromophore from a good fluorophore to a good photothermal converter.^8^ Based on this knowledge, we designed BSA-stabilized PFP nanodroplets incorporating porphyrins with perfluorinated chains to assess: (i) the effect of elevated temperatures on BSA denaturation and on the stability of nanodroplets, (ii) the coalescence of nanodroplets with fluorescein- and rhodamine-labelled BSA, (iii) the gain in volume expansion due to nanodroplet vaporization relative to that due to thermoelastic expansion for the same laser fluences, (vi) the feasibility of producing nanodroplets with low vaporization thresholds that undergo reversible condensations thousands of times with large and reproducible transient volume changes each time.

Laser fluence limits for human skin exposure depend on wavelength, but for nanosecond lasers emitting in the PAT spectral region, they range from ∼20 mJ cm^−2^ at 700 nm to ∼80 mJ cm^−2^ at 1000 nm.^32^ Moreover, light attenuation by human tissues may reduce light fluence to ∼1 mJ cm^−2^ at a depth of ∼1 cm.^33^ These restrictions emphasize the importance of low vaporization thresholds in phase-change contrast agents. Moreover, PAT relies on data collection for a few seconds or minutes at 10 to 20 Hz, and the photoacoustic signal must remain stable for thousands of laser pulses. Our design of PFP droplets intends to reconcile low vaporization thresholds with fast recondensation. Admittedly, paramagnetic complexes of tetraphenylporphyrins, which have electronic absorptions in the 550–600 nm range, are in the lower limit of interest in PAT due to competitive absorption by endogenous chromophores. These porphyrins are nevertheless interesting because they are the direct precursors of the corresponding bacteriochlorins, whose molar absorption coefficients at 829 nm can reach *ε*_829_ = 4.5 × 10^4^ M^−1^ cm^−1^.^34^ We show that PFP droplets stabilized by labelled BSA and incorporating fluorinated porphyrins are very stable, do not coalesce, undergo reversible explosive vaporization for laser fluences above 2.5 mJ cm^−2^ and repeatedly generate PAWs one order of magnitude higher than thermoelastic expansion alone.

## Results and discussion

### Synthesis and characterization of fluorinated *meso*-substituted porphyrins

**Fig. 1:**
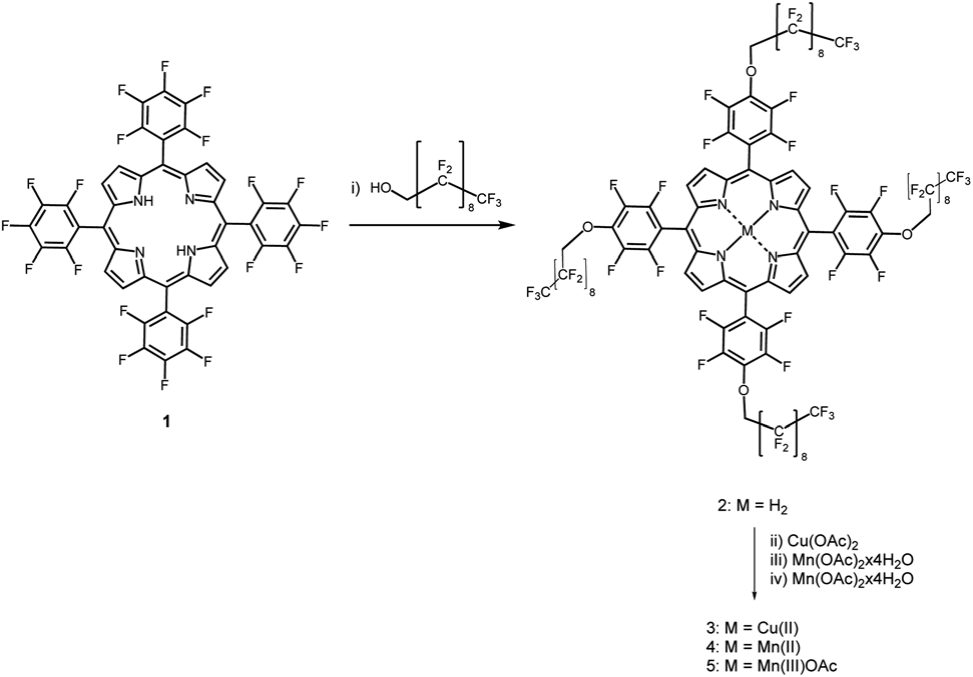
Synthetic route for the preparation of the fluorinated porphyrins. Reagents and conditions: (i) DMF, NaH, 50 °C, 48 h; (ii) CuCl_2_, DME, 50 °C, 1 h; (iii) Mn(OAc)_2_·4H_2_O, DME/THF, 2,6-lutidine, argon atmosphere, 80 °C, 48 h; (iv) Mn(OAc)_2_·4H_2_O, DMF, 2,6-lutidine, argon atmosphere, 140 °C, 48 h, then air atmosphere, room temperature, 3H_2_SO_4_ drops.

The route adopted for the synthesis of the fluorinated porphyrins is depicted in Fig. 1. 5,10,15,20-(pentafluorophenyl)porphyrin (1) was used as platform to obtain free base porphyrin 2 following slight modifications of the literature.^35,36^ Through nucleophilic substitution reaction, 1 was reacted with 1*H*,1*H*-perfluorodecan-1-ol as nucleophile, in the presence of NaH as base and *N*,*N*-dimethylformamide (DMF) as solvent. After purification by column chromatography, product 2 was obtained in 50% isolated yield and characterized by ^1^H, ^19^F NMR spectroscopy and ESI-FIA-TOF mass spectrometry (Fig. S1–S4†). The formation of tetra-substituted porphyrin 2 is corroborated by the loss of the characteristic triplet (*p*-F) ^19^F NMR signal at around −150 ppm, when compared with the ^19^F NMR spectrum of 1, which presents its three typical (*m*-F), (*p*-F) and (*o*-F) signals at −160.2, −150.1 and −135.4 ppm.

Then, complexation of porphyrin 2 with copper and manganese salts afforded the corresponding metal complexes, namely the copper(ii), manganese(ii) and manganese(iii) complexes 3, 4 and 5, respectively, in 85%, 85% and 80% yields. Particularly, the Mn(ii) porphyrin complex 4 could be obtained using manganese diacetate under inert atmosphere, while its induced oxidation under air atmosphere aided by acidic conditions afforded the Mn(iii) complex 5. The Mn(ii) complex was particularly stable due to the presence of the highly electronegative fluorinated phenyl groups, as observed earlier.^36^ The paramagnetic complexes were structurally characterized by their mass spectra (Fig. S4–S7†).

### Properties of fluorinated *meso*-substituted porphyrins

**Fig. 2:**
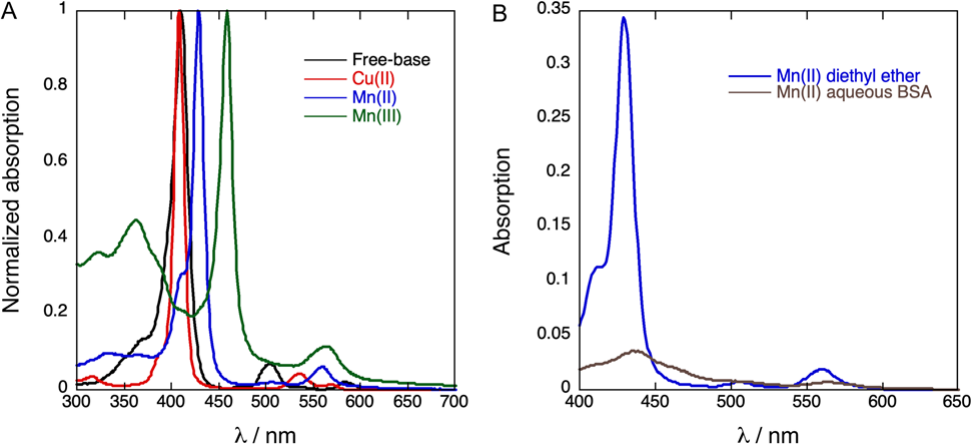
(A) Normalized absorption spectra of 2 (free-base porphyrin), 3 (Cu(ii)porphyrin), 4 (Mn(ii)porphyrin) and 5 (Mn(iii)porphyrin) in diethyl ether. (B) Absorption spectra of Mn(ii)porphyrin in diethyl ether (blue, 4-DEE) and in aqueous BSA (brown, 4-BSA).

Metalation of fluorinated free-base porphyrin 2 changes UV-vis absorption spectra very much the same way as observed in the complexation of other free-base tetraphenylporphyrins with Cu(ii), Mn(ii) or Mn(iii) ions,^37^Fig. 2A. Cu(ii) complexation leads to a small hypsochromic shift of the Soret band, from 410 nm in the free-base porphyrin 2 to 408 nm in the Cu(ii) complex 3, similar to that of tetrakis(perfluorophenyl)porphyrins.^38^ The Mn(iii) complex 5 exhibits a large bathochromic shift of the Soret band to 459 nm, comparable to that observed for Mn(iii) tetraphenylporphyrin.^34^ The corresponding bathochromic shift of the Soret band of the Mn(ii) complex 4 is much smaller. The molar absorption coefficients of the porphyrins are presented in Table 1.

**Table 1:** Soret band molar absorption coefficient of porphyrins in diethyl ether

	Soret peak/(nm)	*ε*/(M^−1^ cm^−1^)
2: Free-base	410 nm	2.63 × 10^5^
3: Cu(ii)	408 nm	4.26 × 10^5^
4: Mn(ii)	428 nm	9.10 × 10^4^
5: Mn(iii)	458 nm	1.16 × 10^5^

We were unable to dissolve measurable amounts of 3 in aqueous BSA and this copper derivative was not further investigated. Fig. 2B shows that the Soret band of 4 at 438 nm in aqueous BSA is broadened and has a bathochromic shift relative to diethyl ether. This indicates that 4 has some degree of aggregation in aqueous BSA in the low micromolar range. It was also possible to obtain an absorption spectrum of 5 in aqueous BSA (Fig. S8†), but it proved to be a mixture of Mn(iii) and Mn(ii). Mn(ii) porphyrins are unstable towards aerial oxidation and are readily oxidized to the corresponding Mn(iii) porphyrins.^39^ However, the electron-withdrawing effect of fluorine substituents in the phenyl ring and in the appended chains is expected to increase the oxidation potential of 4 and stabilize this Mn(ii)porphyrin against oxidation. This explains the observation of a mixture of 4 and 5 in aqueous BSA when an aqueous BSA of 5 is prepared. In view of the higher solubility and higher stability of 4 we focused our work on the incorporation of 4 in PFP droplets.

Long fluorinated chains such as those present in our porphyrins tend to increase solubility in fluorinated solvents but solubility in linear perfluorinated alkanes, such as PFP, remains a challenge.^31^ The faint color of 4 in PFP solution indicates that this fluorinated porphyrin is sparingly soluble in linear perfluorinated alkanes. In order to incorporate 4 in BSA shells of PFP droplets dispersed in water, we added a concentrated diethyl ether solution of 4 to 2 mg mL^−1^ BSA in water (1 : 50, v/v), added PFP (1 : 9) and then agitated the mixture vigorously in a vortex mixer and an ultrasound bath. The result, 4-BSA-PFP, is a strongly scattering emulsion.

### Effect of elevated temperatures on BSA denaturation and on the stability of nanodroplets

**Fig. 3:**
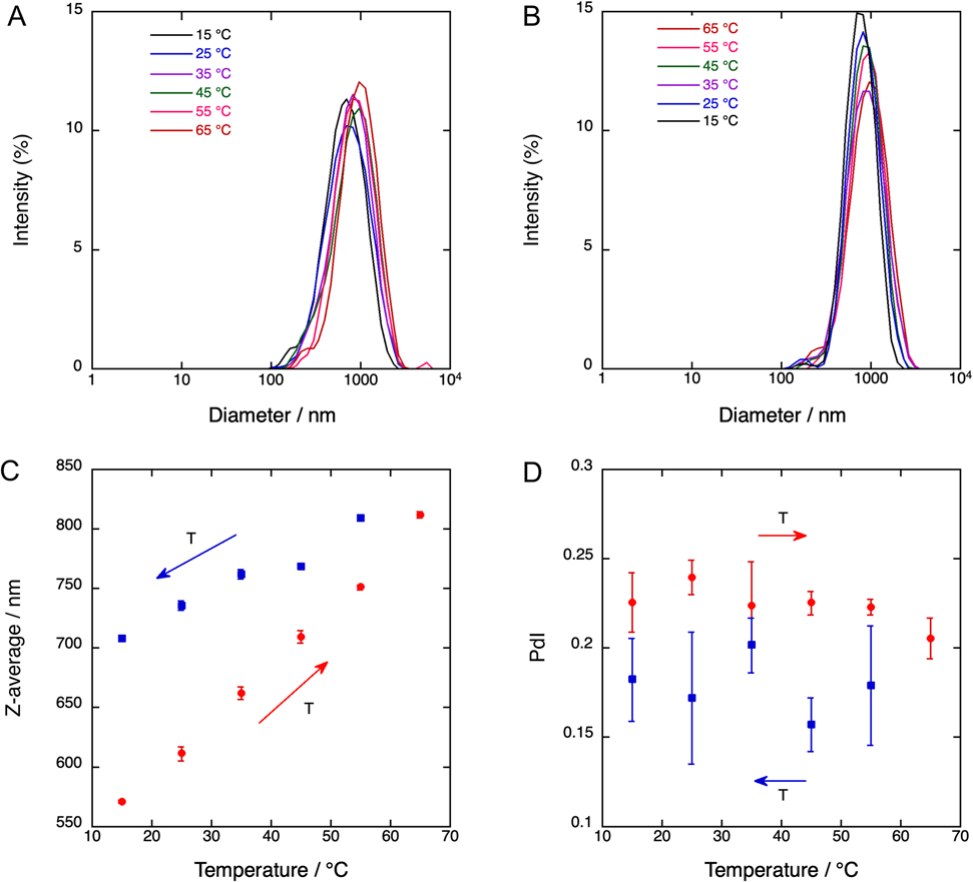
Particle size distribution of Mn(ii)-porphyrin droplets, 4-BSA-PFP, prepared with 150 s of sonication of a 1.7 mL solution. (A) Size distribution with increasing temperatures. (B) Size distribution with decreasing temperatures. (C) Dependence of size on temperature. (D) Polydispersity indexes (PdI) for each measurement. The Z-average and PdIs are averages of three consecutive measurements at the same temperature.

The sizes of 4-BSA-PFP droplets, measured by DLS, depend on sonication times and temperature. Fig. S9† shows that 30 s of sonication of a 1.7 mL solution warming from an ice bath 25 °C produces 728 ± 51 nm diameter droplets, 60 s of additional sonication at 25 °C reduce the average diameter to 657 ± 26 nm, and 30 s plus additional 120 s at 25 °C yields particles with 441 ± 13 nm in diameter. Fig. 3 shows that the average particle size also depends on the temperature, increasing from 570 nm at 15 °C to 810 nm at 65 °C, in a sealed quartz cuvette. The polydispersity indexes (PdI) of these emulsions are relatively small. After each temperature increase, the sample was stabilized for 60 s before DLS measurements. Upon colling to room temperature there is a small decrease in size but the nanodroplets remain larger than their original size at room temperature. Fig. S10–S11† show that a similar behavior was found for 2-BSA-PFP and 5-BSA-PFP droplets.

It is known that the vaporization threshold decreases both with an increase in temperature and with an increase in droplet diameter.^40^ The nanodroplets size influences the vaporization process because smaller nanodroplets have higher core boiling points due to increased pressure.^41,42^ Vaporization temperatures above 70 °C are expected for PFP droplets with sub-micrometer radii.^40,43^ In view of the distribution of sizes shown in Fig. 3, we expect 4-BSA-PFP droplets to be stable at room and body temperatures. BSA denaturates and aggregates with commensurable rates at temperatures above 60 °C in the concentration range of our experiments.^44^ However, a water-soluble porphyrin derivative bound to BSA and subject to laser pulses did not change the conformation of BSA.^29^ BSA is likely to be a minor contributor to the difference in average sizes at room temperature between the nanodroplets kept at room temperature and those heated to 65 °C. PFP droplets with BSA shells proved quite resilient to temperature changes and may withstand the local temperature increase inherent to the vaporization process.

### Evaluation of the coalescence of nanodroplets using labelled BSA

**Fig. 4:**
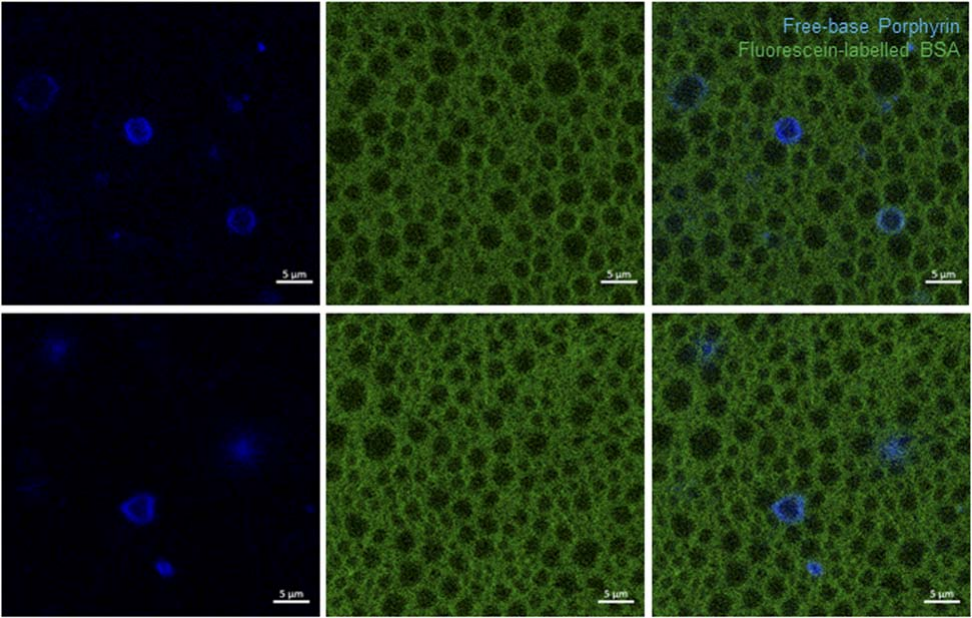
Confocal microscopy of PFP structures loaded with free-base porphyrin 2 (blue color) in aqueous fluorescein-labelled BSA (green color), 2-BSA-PFP. Two fields of view are presented. The porphyrin was excited at 405 nm and its fluorescence collected at 605/70 nm. Fluorescein-labelled BSA was excited at 488 nm and its fluorescence collected at 525/50 nm. Scale bar is 5 μm.

Further insight into the structure of our 4-BSA-PFP system was obtained using confocal microscopy. We used fluorescein-labelled BSA and the fluorinated free-base porphyrin 2 to visualize the localization of BSA and of 2 in the structure. The replacement of 4 by 2 is required because paramagnetic porphyrins have extremely short lifetimes and negligible fluorescence. This replacement does not significantly change the properties of the nanodroplets, as shown in Fig. S10.†Fig. 4 presents the confocal microscopy of 2-BSA-PFP in aqueous solution using fluorescein-labelled BSA, where the fluorescence in green comes from fluorescein and in blue comes from 2. Fig. S12† presents confocal microscopy of 4-BSA-PFP, where no fluorescence is observed from 4, as expected.

The free-base porphyrin 2 was not incorporated in all the structures. It fluoresces in the periphery of the darker circular structures, with diameters in the 1–5 μm range, wherein PFP is certainly concentrated. Fluorescein-labelled BSA fluorescence is also absent from the PFP microphase. It gives a green background to the aqueous solution. Fig. S13† presents additional images of 2-BSA-PFP at various dilution factors. Dilution with water reduces the green background but the BSA shell remains clearly visible, as well as the fluorescence patterns of 2. This suggests that the BSA shell is a very stable structure but at the BSA concentrations employed ([BSA] ≈ 30 μM), some BSA is in the bulk of the aqueous solution.

**Fig. 5:**
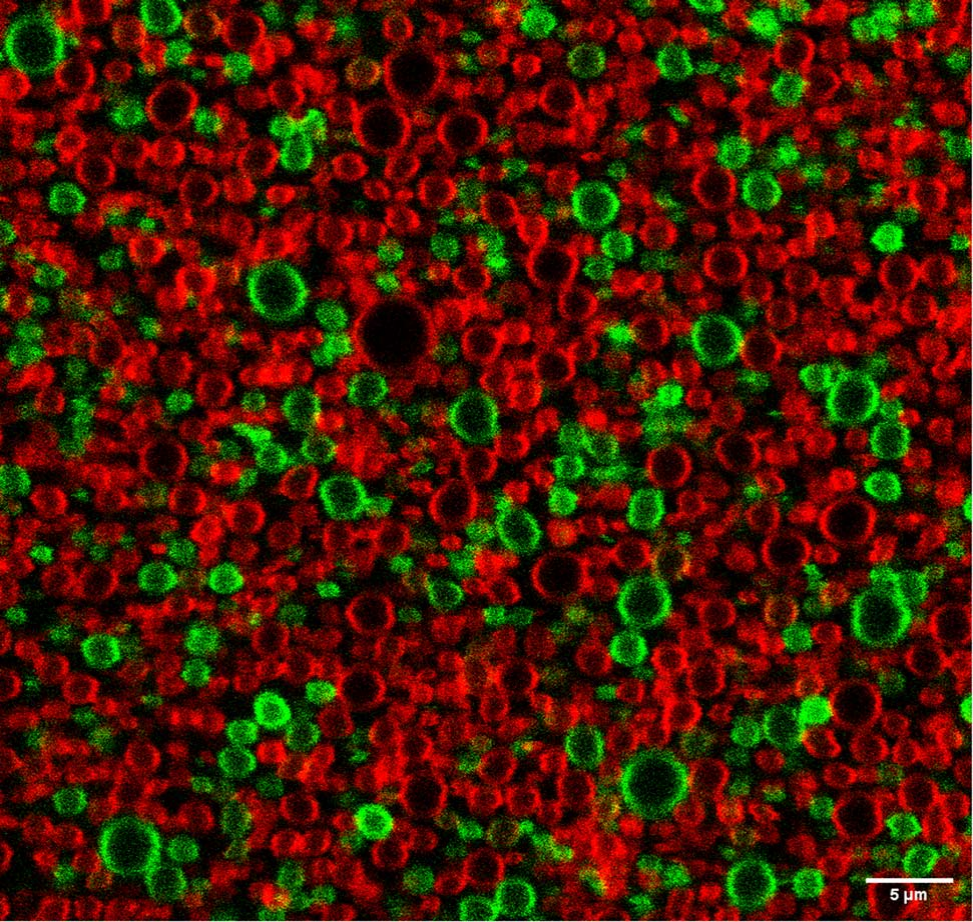
Confocal microscopy of PFP structures in aqueous fluorescein-labelled BSA (green) and rhodamine-labelled BSA (red). Fluorescein-labelled BSA was excited at 488 nm and its fluorescence collected at 525/550 nm. Rhodamine-labelled BSA was excited at 555 nm and its fluorescence collected at 605/670 nm. Scale bar is 5 μm.

Additional evidence for the high stability of this shell, absence of droplet coalescence and slow exchange dynamics between BSA molecules in the shell and the bulk of the aqueous solution, was obtained using a mixture of nanodroplets prepared with fluorescein-labelled or rhodamine-labelled BSA. Fig. 5 shows that the droplets maintain their labels after mixing the two populations because we do not see droplets with a mixture of fluorescein and rhodamine labels. Coalescence of the droplets should give a mixture of green and red labels. Fig. S14† shows various fields of view of droplets at room temperature that were kept on ice, at room temperature or heated to 65 °C for one hour. They have essentially the same features as described above, although the heat-treated droplets present more homogeneous patterns.

The spatial resolution of confocal microscopy is not sufficient to allow for proper visualization of nanodroplets with sizes below 1 μm. Moreover, larger nanodroplets are heavier and tend to accumulate at the deeper layers of the wells, which means that the choice of the focus plane also impacts on the distribution of sizes visualized. Fig. 4 and S13† show solid and ring patterns of the fluorescence of porphyrin 2. It is tempting to assign the solid fluorescence pattern of 2 to droplets incorporating the porphyrin and the ring fluorescence pattern of 2 to microbubbles with a porphyrin shell. This has been done in the literature for other dyes but we are inclined to an alternative explanation. The focus plane of confocal microscopy more easily finds the center of a 2–3 micron-sized droplet than of a smaller droplet. When this plane intersects the middle of a larger droplet, the fluorescence from 2 has a ring pattern. However, if the droplet is smaller, or if the droplet is slightly above or below the focus plane and looks smaller, the image obtained has a pattern of solid fluorescence. This may give the impression that 2 is inside smaller (nano)droplets and in the shell of larger (micro)bubbles, but it is more likely that 2 is always in the BSA shell. The same applies to BSA but only becomes obvious for more dilute solutions, with lower fluorescence background of the BSA labels, Fig. S13.† The illustration of various and large fields of view, rather than one structure of interest, is important to picture the diversity of these emulsions and make a proper interpretation of the data.

Taken together, these data show that albumin forms a stable shell around PFP domains, excess BSA is dissolved in the bulk aqueous solution, free-base porphyrin 2 is located in PFP shells and coalescence of the droplets does not occur in our experimental conditions.

### Volume changes in nanodroplet vaporization and recondensation

The amplitude of PAWs generated by thermoelastic expansion (*P*_th_) after absorption of a short laser pulse is proportional to the heat deposited in the medium (*H*) and to the thermoelastic properties of the medium^45^2
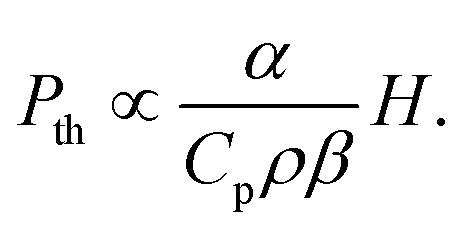
In materials, this dependence is often expressed in terms of the Grüneisen parameter *Γ* = *α*/(*C*_p_*ρβ*). Eqn (2) is closely followed in dilute homogenous solutions. The low thermal expansion coefficient of water is largely responsible for the smallness of thermoelastic expansions in this solvent, which tend to zero at 3.9 °C.^46^

**Fig. 6:**
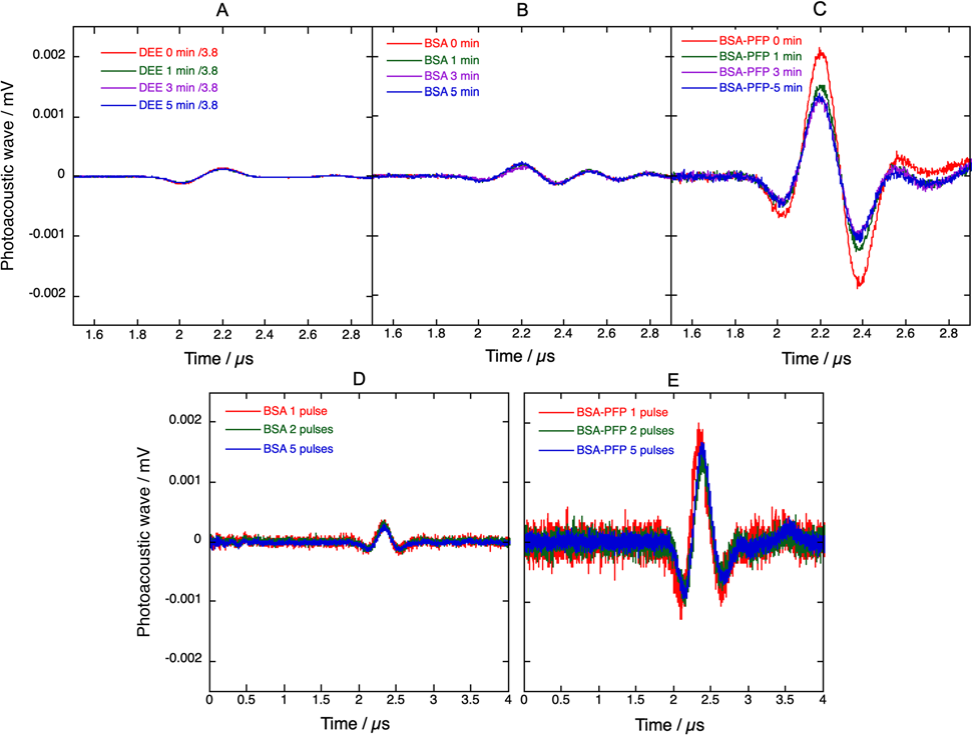
Photoacoustic waves generated by pulsed laser excitation at 438 nm (∼0.5 mJ per pulse, 20 Hz repetition rate) of 4 in different media. Average of 50 photoacoustic waves measured initially and after 1, 3 and 5 min of continuous excitation of 4 in (A) diethyl ether, DEE, (B) aqueous BSA and (C) PFP droplets in aqueous BSA, BSA-PFP. PAWs in DEE were normalized by the ratio of aqueous BSA/DEE absorption at 438 nm. PAWs generated with the first laser pulse, the first 2 laser pulses or the first 5 laser pulses are presented in (D) for 4-BSA and (E) for 4-BSA-PFP. 4-BSA and 4-BSA-PFP were prepared from the same amount of stock solution of 4 in diethyl ether, but the absorption of 4-DEE is 3.8× higher than of 4-BSA.

Fig. 6 presents PAW generated when 4 absorbs laser pulses with a fluence of ∼3 mJ cm^−2^ at 438 nm in 4-BSA solutions or in 4-BSA-PFP emulsions, prepared from the same volume of a stock solution of 4 in diethyl ether. The PAWs generated by 4 in diethyl ether (DEE) presented in Fig. 6 were normalized by the ratio of absorption of 4 in aqueous BSA and DEE at 438 nm (1/3.8 from Fig. 2B). By molecular design, all the optical energy absorbed by 4 is rapidly lost by radiationless processes and the PAWs in diethyl ether and aqueous BSA solutions should be entirely due to thermoelastic expansion. Surprisingly, the absorption-normalized PAWs intensities in 4-DEE are slightly lower than in aqueous BSA. This is the opposite of what is expected from the thermoelastic properties of water and diethyl ether. Using the data for water^45^ and for diethyl ether, eqn (2) predicts 8.7× higher peak pressures in diethyl ether than in pure water. The similarity between normalized PAWs in 4-DEE and 4-BSA can be rationalized considering that 4 is insoluble in water and that it is necessarily associated with BSA in aqueous media. The source of the photoacoustic signal in 4-BSA is the response of BSA to sudden heat deposition after the absorption of the laser pulse by 4.

The higher amplitudes of PAWs in 4-BSA-PFP emulsions cannot be assigned to thermoelastic expansion of PFP because eqn (2) with the thermoelastic properties of PFP (*α* = 2 × 10^−3^ K^−1^, *C*_p_ = 0.156 cal g^−1^ K^−1^, *ρ* = 1.627 g cm^−3^, *β* = 2.74 × 10^−10^ cm^2^ dyn^−1^)^47,48^ predicts PAW amplitudes in PFP half the size of those in DEE. Hence, the amplification must be associated with a dramatic increase in structural volume.

The ratio between the radius of a nanodroplet (*r*_l_) and the radius of a microbubble (*r*_g_) can be estimated from3
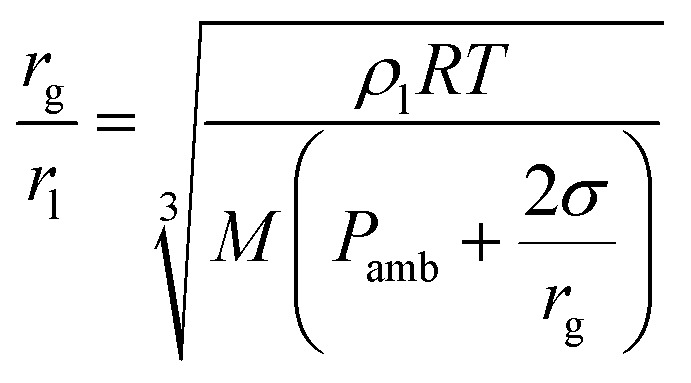
where *ρ*_l_ is the liquid density (1.63 g cm^−3^ for PFP), *M* is the molecular weight (*M* = 288 g mol^−1^ for PFP), the ambient pressure is *P*_amb_ = 101 kPa, and *σ* is the surface tension (*σ* ≈ 30 mN m^−1^).^40,43^ Evaluation of the equation above gives an estimate of *r*_g_ = 2 μm when *r*_l_ = 420 nm is employed. Hence, vaporization of a liquid nanodroplet should increase the structural volume by two orders of magnitude.

Fig. 6 shows that the intensity of the PAWs generated in 4-BSA-PFP emulsions is 10× higher than those generated in 4-BSA for the same amount of 4 and the same laser fluence. This supports a mechanism of explosive vaporization of nanodroplets concomitant with the absorption of the laser pulse. The microbubbles formed may re-condensate and regenerate nanodroplets or may coalesce and escape the irradiated volume before the next laser pulse is absorbed. In the first case, the PAW generated by subsequent laser pulses may reproduce the intensity of the first PAW, whereas in the second case the intensity of the PAW should decrease and tend to the value observed for 4-BSA. Fig. 6D and E shows that the average of the PAWs generated by two laser pulses, is similar to that of the first laser pulse, and the same applies for 5 and 50 averages. Fresh PFP droplets solutions were employed in each experiment. This suggests that the microbubbles can re-condensate and that the recondensation dynamics is fast compared with laser repetition rate of 10 Hz. Thus, phase transition of PFP nanodroplets amplify photoacoustic signals and the amplification is stable. This stability allowed for the measurement of the photoacoustic spectrum of 4-BSA-PFP (Fig. S15†), which corresponds well with the electronic absorption spectrum of 4-BSA.

### Vaporization threshold and efficiency

**Fig. 7:**
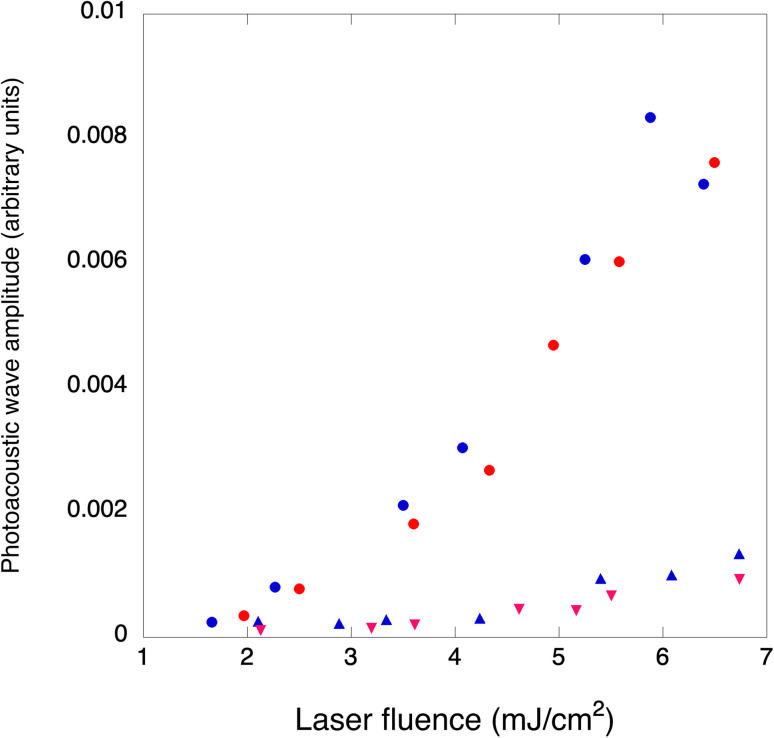
Photoacoustic waves amplitudes as a function of laser fluence, for the same amount of 4 in 4-BSA (triangles) and 4-BSA-PFP (circles). Blue: samples kept at room temperature. Red: samples headed to 65 °C for one hour and cooled to room temperature before the measurements.

PAWs due to thermoelastic processes show linear responses with the laser energy in homogenous solutions. Although two-photon absorption may increase the amplitude of the observed waves for very high laser fluences,^49^ this is not relevant for imaging organisms. It was recently emphasized that PAWs generated in the vaporization of nanodroplets incorporating dyes have a supra-linear dependence on laser fluence and that this may be used for increased contrast in imaging applications.^14,16^ We investigated laser fluence dependences of PAWs generated by 4-BSA and 4-BSA-PFP at room temperature to determine PFP vaporization thresholds. Two different conditions were tested while maintaining the same amount of 4. In one condition, PAWs were measured for increasing laser fluences without prior heating of the samples. In the other condition, samples were heated to 65 °C for one hour, allowed to cool to room temperature and PAWs were again measured for increasing laser fluences. Fig. 7 shows that heating and then cooling down to room temperature leads to a minor decrease in the amplitude of the photoacoustic waves, which means that very few nanodroplets were lost in this pre-conditioning process. At low laser fluences (<2 mJ cm^−2^) PAWs generated by 4-BSA and by 4-BSA-PFP are equally small. However, we observed an order-of-magnitude increase in the photoacoustic waves generated by 4-BSA-PFP relative to 4-BSA for laser fluences >3 mJ cm^−2^. Vaporization requires that local heating of the droplets increases local temperature above the threshold for phase transition. This depends on the laser fluence, on the absorption of optical energy by the dye, on its efficiency of optical to thermal energy conversion and heat deposit in the liquid core. The discontinuity observed in Fig. 7 at ∼2.5 mJ cm^−2^ indicates that this laser fluence provided enough local heating for vaporization.

Vaporization thresholds also depend on the nature of the nanodroplets core and shell. For example, C_3_F_8_, C_4_F_10_ and C_5_F_12_ nanodroplets with lipid shells have vaporization thresholds of 10.6 ± 5.1, 26.1 ± 21.7 and 97 + 20 mJ cm^−2^, respectively.^50^ Similar results were obtained with C_4_F_10_, C_5_F_12_ and C_6_F_14_ nanodroplets with polymer shells, which exhibited vaporization thresholds of 11, 20 and 70 mJ cm^−2^, respectively.^19^ These sequences highlight the correlation between vaporization thresholds and boiling points of the core materials. C_6_F_14_ nanodroplets shells made with a fluorosurfactant, lipids or BSA show increasing vaporization thresholds, in the 15 to 50 mJ cm^−2^ range, that seem to correlate with interfacial tensions at the water:nanodroplet interfaces.^28^ The relatively low vaporization threshold of our system, ∼2.5 mJ cm^−2^, suggests an interfacial tension much lower than that usually enabled by BSA. The four perfluorinated chains of the porphyrin strongly bound to BSA may originate a kind of a fluorosurfactant that lowers interfacial tension and, consequently, the boiling temperature of the nanodroplets.

It is interesting to consider how many nanodroplets need to be vaporized per laser pulse to yield the observed PAW amplification. As discussed above, the amplitudes of PAW in DEE and aqueous BSA are similar, which allow for the calculation of Δ*V*_th_ = 6.5 × 10^−7^ cm^3^ using the thermoelastic parameters of diethyl ether, *E*_L_ = 0.5 mJ and *ϕ*_th_ = 1. Considering the volume of PFP suspended in the volume of the emulsion and an average diameter of 900 nm for the nanodroplets, we estimate to have 8 × 10^8^ droplets per mL and 10^6^ droplets in the irradiated volume. As shown in Fig. 4 and further illustrated by other droplets in Fig. S13,† only a fraction of the droplets is colored with dye molecules. Hence, we roughly estimate to have 10^5^ photoactivatable droplets in the excited volume. The volume change with the vaporization of each droplet can be estimated from eqn (3) and is approximately equal to the volume of the microbubbles, Δ*V*_st_ = 4 × 10^−11^ cm^−3^ per particle. In order to obtain Δ*V*_st_ = 6.5 × 10^−6^ cm^3^ and explain the order of magnitude increase of 4-BSA-PFP with respect to 4-BSA, we need ∼10^5^ droplets to vaporize, which is approximately the number of photoactivatable droplets in the irradiated volume. There are various assumptions in these rough calculations, but they do indicate that the efficiency of vaporization process (*ϕ*_st_) is very high for laser fluences >2.5 mJ cm^−2^.

## Conclusions

Confocal microscopy of fluorescein- and rhodamine-labelled BSA and of fluorescent porphyrins, together with dynamic light scattering, allowed for a detailed characterization of PFP droplets dispersed in aqueous BSA. We observed a distribution of sizes that depends on the sonication time. The study of droplets with diameters in the 0.5–5 μm range reveals that the PFP core is surrounded by a shell of BSA. This shell is remarkably stable but it is possible that some BSA is also present in the bulk aqueous solution at high BSA concentrations. Highly fluorinated porphyrins are preferentially located in the BSA shell around the PFP droplets. Pulsed laser excitation of these porphyrins at fluences higher than 2.5 mJ cm^−2^ produces efficient explosive vaporization of the nanodroplets. The linear C_8_F_17_ fluorinated chains introduced in fluorophenylporphyrins were not sufficient fluorous to color the majority of the nanodroplets, but lowered the vaporization threshold to one of the lowest reported for PFP. The microbubbles thus generated rapidly recondense to nanodroplets, which allows for the repeated generation of amplified photoacoustic waves with subsequent laser pulses. Recondensation requires nanodroplet stability that is usually associated with high boiling points and high vaporization thresholds, but is achieved here at low vaporization thresholds. This is interpreted as 4-BSA behaving as a surfactant with a perfluorinated tail. Incorporation of dye molecules in 10% of the PFP droplets shells increases the amplitude of the photoacoustic waves by one order of magnitude relative to the same amount of dye molecules in aqueous BSA. Phase-transition contrast agents with fluorous dyes have the potential to increase the sensitivity of photoacoustic detection by two orders of magnitude with respect to contrast agents based on thermoelastic expansion, and may yield reproducible photoacoustic signals with laser fluences and laser repetition rates of clinical relevance.

## Experimental procedures

### General methods

Commercially available reagents (Aldrich and Fluorochem suppliers) were used as received. All solvents were pre-dried according to standard laboratory techniques. UV-visible absorption spectra were recorded on a Hitachi U-2010 Lambda 25 UV/vis Spectrophotometer (PerkinElmer) in the range 300–800 nm using quartz cells. The molar absorption coefficients were determined using diethyl ether as solvent. ^1^H-NMR and ^19^F-NMR spectra were recorded on a 400 MHz Bruker Avance III NMR spectrometer (400.101 and 376.5 MHz respectively). Proton chemical shifts are given in parts per million (ppm) relative to tetramethylsilane at *δ* 0.00 ppm and ^19^F relative to trifluoroacetic acid at *δ* −76.55 ppm. Mass spectra (ESI-FIA-TOF) were acquired using an Applied Biosystems Voyager DE-STR instrument equipped with a nitrogen laser (*λ* = 337 nm). Dynamic Light Scattering (DLS) measurements were done on a Zetasizer Nano ZS (Malvern). Each sample was equilibrated for 60 s at the desired temperature (15 to 65 °C) and each measurement was an average of at least 12 runs. Three consecutive measurements were performed for each sample to evaluate its stability. The samples were measured in quartz cells. The results were analyzed using the equipment software Zetasizer Software (version 8.00.4831). The average size of the particles was taken from the Z-average size. Confocal microscopy was performed at the MICC Imaging facility of the Centre for Neuroscience and Cell Biology (Coimbra, Portugal) using a Carl Zeiss, LSM 710 microscope. The images were manually selected to obtain a region of interest in a confocal plane. The images collected with the ZEN software were studied with the Image J software.

**Fig. 8:**
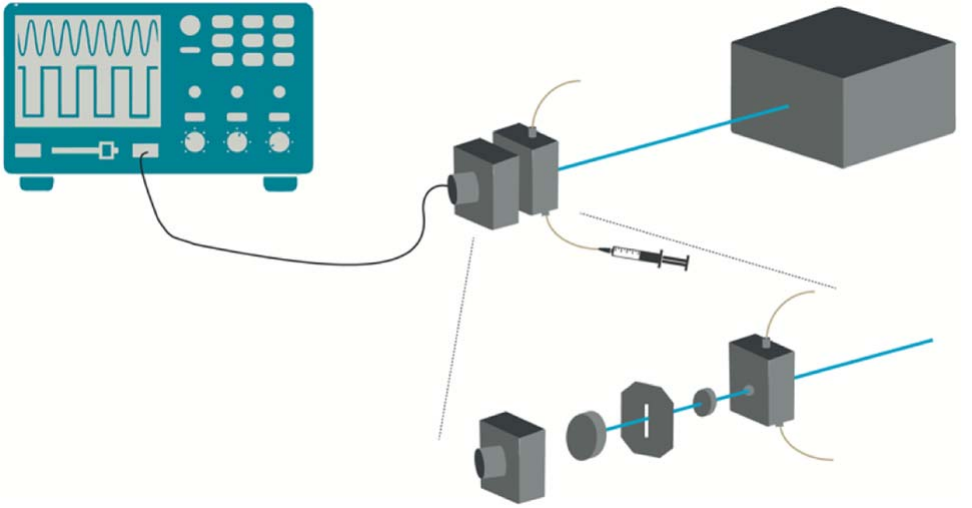
Photoacoustic cell. The spacer between the quartz window and the front-face dielectric mirror is a copper foil 0.1 mm thick. The ultrasonic transducer is coupled to the back of the mirror with a thin layer of silicone. The cell is temperature-controlled with a flow of water through channels drilled in the cell body (not shown for clarity) and connected to a thermostatic bath.

The photoacoustic experiments were performed in a homemade apparatus following the front-face irradiation design described by Arnaut *et al.*,^45^Fig. 8. Pulsed laser excitation at distinct wavelengths employed an OPO (EKSPLA PG 122/SH) pumped by the third harmonic of a nanosecond Q-switched Nd:YAG laser (EKSPLA NL301G). This system delivers ∼6 ns laser pulses with energies between 0.2 and 2.0 mJ per pulse at an adjustable repetition rate in the 0.1–20 Hz range. The detection of the photoacoustic waves employed a 2.25 MHz contact transducer (Panametrics, Olympus). The temperature-controlled photoacoustic cell is composed of an aluminium structure that holds a quartz window pressed against a dielectric mirror, where a transducer is coupled. Channels were drilled in the body of the cell to allow for a temperature-controlled fluid to flow in the cell body and control its temperature. A copper foil placed between the front window and the back mirror determines the optical path, which is 0.1 mm in this work. The test solution was manually injected in the bottom part of the cell using a plastic syringe. The data was collected using an oscilloscope Tektronix model DPO7254.

### Synthesis of free-base fluorinated *meso*-substituted porphyrin 2

A mixture of 5,10,15,20-(pentafluorophenyl)porphyrin (140 mg, 0.14 mmol) and 1*H*,1*H*-perfluorodecan-1-ol (560 mg, 1.12 mmol) was dissolved in 8 mL of dry DMF. After the addition of NaH (26.9 mg, 1.12 mmol) the reaction was stirred at 50 °C under inert atmosphere. After 24 h, more 1*H*,1*H*-perfluorodecan-1-ol (260 mg, 0.56 mmol) and NaH (13.5 mg, 0.56 mmol) were added and the reaction proceeded for another 24 h, until complete consumption of the starting porphyrin, checked by TLC. The reaction mixture was then precipitated with methanol. Purification by column chromatography (silica gel, *n*-hexane:dichloromethane 2 : 1 as eluent) afforded porphyrin 2 in 50% yield as a violet sticky solid. ^1^H NMR (400 MHz, (CD_3_)_2_CO) *δ*, ppm: 8.47 (s, 8H, β-H), 4,54 (t, 8H, OCH_2_), −3.74 (s, 2H, inner NH); ^19^F NMR (376 MHz, (CD_3_)_2_CO) *δ*, ppm: −81.60 (m, 12F (CF_3_)), −120,77 to −126,85 (m, 64F (CF_2_)), −140.88 (m, 8F (*o*-F)), −158.06 (m, 8F (*m*-F)); MS- (ESI-FIA-TOF): *m*/*z* [M + H]^+^ 2894.9899.

### Synthesis of paramagnetic fluorinated porphyrins

#### Copper(ii) complex 3

Free base 2 (33.9 mg, 0.0117 mmol) and copper(ii) chloride (7.87 mg, 0.0585 mmol) were dissolved in 1.5 mL of dimethoxyethane (DME). The reaction was stirred at 50 °C and controlled by UV-vis spectroscopy. After complete conversion (1 hour), the reaction mixture was precipitated with a 2 : 1 methanol : water mixture, and porphyrin 3 was obtained in 85% yield, as a pink solid. MS- (ESI-FIA-TOF): *m*/*z* [M]^+^ 2954.8955.

#### Manganese(ii) complex 4

Free base 2 (33.9 mg, 0.0117 mmol) and manganese(ii) acetate tetrahydrate (14.18 mg, 0.082 mmol) were dissolved in 2 mL of DME with 5 drops of tetrahydrofuran (THF). Then, 2,6-lutidine (4.3 mg, 0.04 mmol) was added and the reaction was stirred at 80 °C in argon atmosphere and controlled by UV-vis spectroscopy. After complete conversion to the manganese complex (48 hours), the reaction mixture was precipitated with a 2 : 1 methanol : water mixture, and porphyrin 4 was obtained in 85% yield, as a brown solid. MS-(ESI-FIA-TOF): *m*/*z* [M-OAc]^+^ 2946.9085.

#### Manganese(iii) complex 5

Free base 2 (65 mg, 0.0224 mmol) and manganese(ii) acetate tetrahydrate (38.43 mg, 0.1568 mmol) were dissolved in 1 mL of DMF. Then, 2,6-lutidine (8.5 mg, 0.08 mmol) was added and the reaction was stirred at 140 °C in argon atmosphere and controlled by UV-vis spectroscopy. After complete conversion to the manganese(ii) complex (48 hours), the inert atmosphere was removed and the reaction was allowed to reach room temperature under air atmosphere. Then, a few drops of sulfuric acid were added to promote the full oxidation to the manganese(iii) complex, observed by UV-vis spectroscopy. The reaction mixture was then precipitated with a 2 : 1 methanol : water mixture, and porphyrin 5 was obtained in 80% yield, as a brown solid. MS-(ESI-FIA-TOF): *m*/*z* [M-OAc]^+^ 2946.9078.

### Synthesis of BSA with covalently linked fluorescent probes

The thiol reactive fluorescein and rhodamine derivatives, fluorescein-5-iodoacetamide (5-IAF) and tetramethylrhodamine-5-iodoacetamide (5-TMRIA), were purchased from Setareh Biotech. The reaction with BSA (Fatty Acid Free Powder, Heat Shock Treated, Fisher Scientific) was performed at pH 8.3 (obtained by mixing PBS at pH 7.4 with 0.2 M bicarbonate adjusted to pH 9 with NaOH, at 20 : 1 v/v, respectively).^51^ BSA was dissolved in the reaction media at 0.8 mg mL^−1^ and equilibrated with 4 mM octanoic acid (Sigma Aldrich) for 30 min, to block BSA binding sites and thus prevent non-covalent binding of fluorescein to BSA. The reactive dye was dissolved in anhydrous DMF (100 μL per mg) and added dropwise up to an equimolar ratio with BSA under gentle agitation. The reaction was allowed to proceed for 2 h at 37 °C in the dark. Unreacted dye was removed by size exclusion using a G-25 Sephadex column and eluted with PBS. The BSA conjugate was aliquoted and stored at −20 °C until use. The number of dye molecules per BSA was calculated from the absorption spectra considering the molar extinction coefficients of the dyes at their lowest energy band, and that of both the dye and BSA at 280 nm,^52,53^ being ∼0.6 for BSA-Fluorescein and ∼0.7 for BSA-Rhodamine, Fig. S16.†

### Preparation of droplets

Droplets were prepared using PFP (*i.e.*, dodecafluoropentane, FluoroMed) as core and BSA as shell material, based on a procedure described for indocyanine-loaded PFP nanodroplets.^15^ A 2 mg mL^−1^ BSA aqueous solution was prepared and kept in an ice bath. To prepare blank droplets, 8.8 mL of the BSA solution and 200 μL of diethyl ether were added to a glass vial. Then, 1 mL of PFP was added to the vial and the solution was kept on ice to prevent the evaporation of PFP (boiling point of 29 °C). Next, the vial was placed in a vortex mixer (Janke & Kunkel Ika-Werk VF2) at a medium speed for 10 s, to produce droplets with sub-millimeter diameters (*i.e.*, nanodroplets). Finally, the vial was sonicated (Elma Transsonic 700/H ultrasound bath) for 3 min while shaken by hand, to better emulsify the solution and produce smaller nanodroplets. Only the suspension was used in the experiments, which corresponded to *ca*. half of the PFP added to the BSA solution. Alternatively, for the observation of the shell by confocal microscopy, BSA labelled with fluorescein or with rhodamine was employed in the preparation of the droplets.

Porphyrin-loaded droplets were prepared from diethyl ether stock solutions of the porphyrins. After the addition of the stock solution to the BSA solution, the mixture was left standing refrigerated for 24 h to allow for the deposition of insoluble material. The supernatant was collected and its absorption spectrum was measured. Next, 1 mL of PFP was added to each porphyrin-BSA solution and the vortex and sonication procedures were performed as above to obtain nanodroplets. The nanodroplets were kept in an ice bath until further use. The porphyrins are identified by a number in bold, the porphyrins in diethyl ether by #-DEE, the porphyrins in aqueous BSA solution by #-BSA and the porphyrins in PFP emulsions in aqueous BSA by #-BSA-PFP.

## Author contributions

LGA designed and supervised the experiments. MIPM and MJFC synthesized the fluorinated porphyrins. MIPM, CDFC and MJM performed dynamic light scattering and supervised confocal microscopy. MIPM and FAS measured the photoacoustic waves MIPM and LGA analyzed the data and prepared the manuscript for submission.

## Conflicts of interest

There are no conflicts to declare.

## Supplementary Material

RA-013-D3RA05639B-s001
