# Supplementary material for: Nanodroplet vaporization with pulsed-laser excitation repeatedly amplifies photoacoustic signals at low vaporization thresholds
Source: RSC Adv. 2023 Dec 1;13(50):35040–9. doi: 10.1039/d3ra05639b (PMC10690495; doi:10.1039/d3ra05639b)
Supplement: RA-013-D3RA05639B-s001 [file RA-013-D3RA05639B-s001.pdf]

Electronic Supplementary Material (ESI) for RSC Advances.
This journal is © The Royal Society of Chemistry 2023

Electronic Supplementary Information

Nanodroplet vaporization with pulsed-laser excitation repeatedly amplify photoacoustic waves

Maria Inês P. Mendes [a,b], Carlos D. F. Coelho, [a] Fábio A. Schaberle [a], Maria João Moreno [a], Mário Calvete [a], Luis G. Arnaut [*a]

[a] CQC-IMS, Chemistry Department, University of Coimbra 3004-535 Coimbra, Portugal

[b]LaserLeap Technologies, Rua Coronel Júlio Veiga Simão, Edifício B, CTCV, S/N, 3025-307 Coimbra, Portugal

Fig. S1. ^{19}F-NMR of 5,10,15,20-(pentafluorophenyl)porphyrin (**1**).

Fig. S2: ^{1}H-NMR of compound **2**.

Fig. S3: ^{19}F-NMR of compound **2**.

Fig. S4. ESI-FIA-TOF mass spectrometry of compound **2**.

Fig. S5. ESI-FIA-TOF mass spectrometry of compound **3**.

Fig. S6. ESI-FIA-TOF mass spectrometry of compound **4**.

Fig. S7. ESI-FIA-TOF mass spectrometry of compound **5**.

Fig. S8. Absorption spectrum obtained after dissolving **5** is aqueous BSA (brown line), and other absorption spectra revealing that **4** is formed from **5** under these conditions.

Fig. S9. Sizes of PFP droplets at 25 °C in a 1.7 mL aqueous BSA solution after 30 sec of sonication (728 nm diameter droplets), 90 sec of sonication (657 nm diameter droplets) and 150 sec (441 nm diameter droplets).

Fig. S10. Temperature dependence of the size of PFP droplets in aqueous BSA loaded with free-base porphyrin **2**. A) Dependence of size on temperature. B) Polydispersity indexes (PdI) for each measurement. The Z-Average and PdIs are averages of three consecutive measurements at the same temperature.

Fig. S11. Temperature dependence of the size of PFP droplets in aqueous BSA loaded with Mn(II)porphyrin **4** and Mn(III)porphyrin **5**. The triangle is the particle diameters after cooling to room temperature.

Fig. S12. Confocal microscopy of PFP structures loaded with Mn(II) porphyrin **4** in aqueous fluorescein-labelled BSA (green color). This porphyrin is not fluorescent. Fluorescein-labelled BSA was excited at 488 nm and its fluorescence collected at 525/50 nm. Scale bar is 5 μm.

Fig. S13. Confocal microscopy of PFP structures loaded with free-base porphyrin **2** (blue color) in aqueous fluorescein-labelled BSA (green color). These emulsions were prepared from a stock solution of **2** in tetrahydrofuran. B is a 5x dilution of the initial emulsion A with the addition of water, and C is a 20x dilution. The porphyrin was excited at 405 nm and its fluorescence collected at 605/70 nm. Fluorescein-labelled BSA was excited at 488 nm and its fluorescence collected at 525/50 nm. Scale bar is 5 μm.

Fig. S14. Confocal microscopy of droplets kept at different temperatures and then allowed to return to room temperature. A) Droplets kept on ice. B) Droplets kept at room temperature. C) Droplets heated to 65 °C for one hour. The lower panels are ampliations of the upper panels.

Fig. S15. Photoacoustic spectrum of **4** in PFP droplets dispersed in aqueous BSA. Each data point is an average of the maximum peak of PAWs produced with 50 laser pulses.

Fig. S16. Absorption and fluorescence spectra of the BSA conjugates.

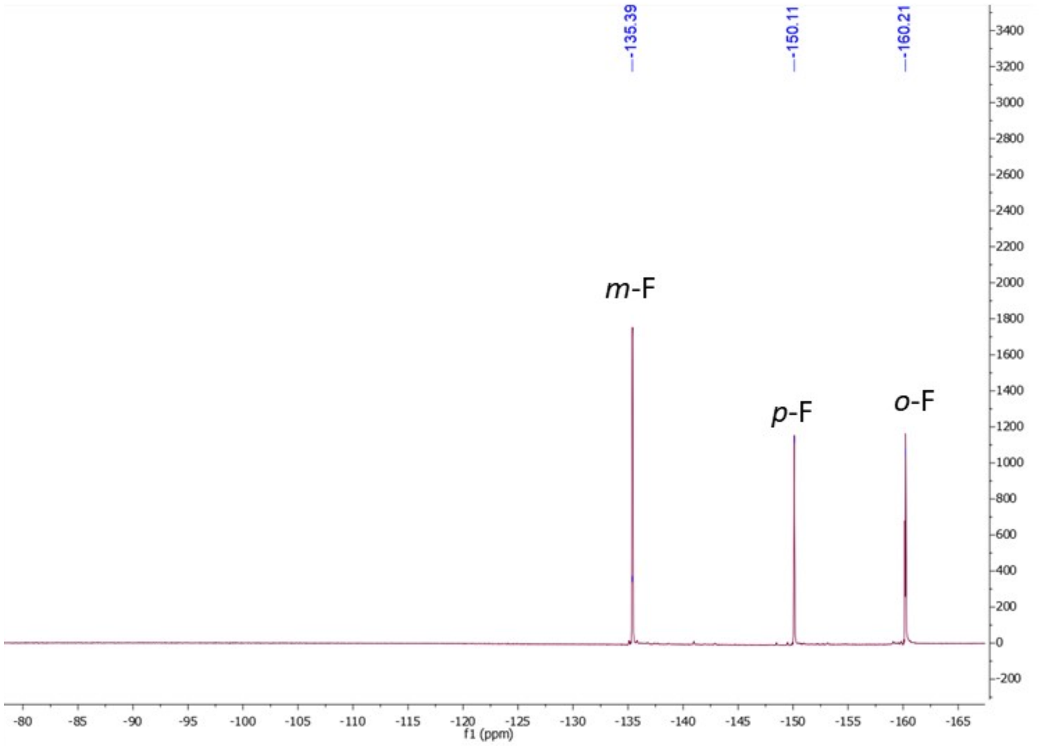

Fig. S1. ^{19}F-NMR of 5,10,15,20-(pentafluorophenyl)porphyrin (**1**)

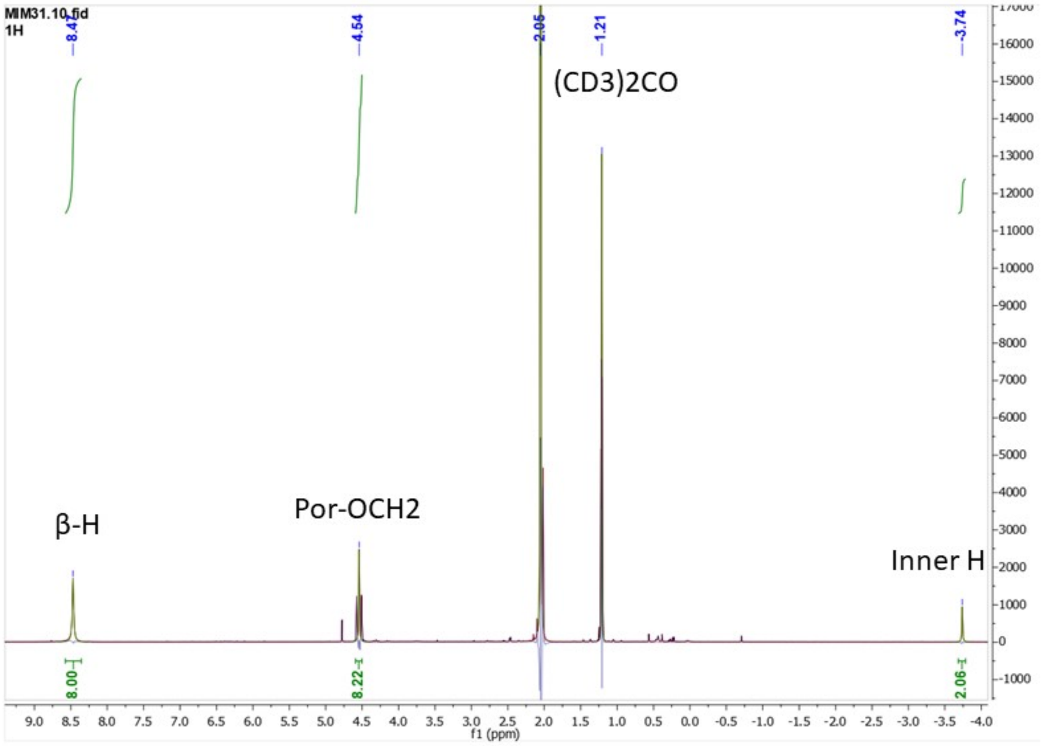

Fig. S2: ^{1}H-NMR of compound **2**.

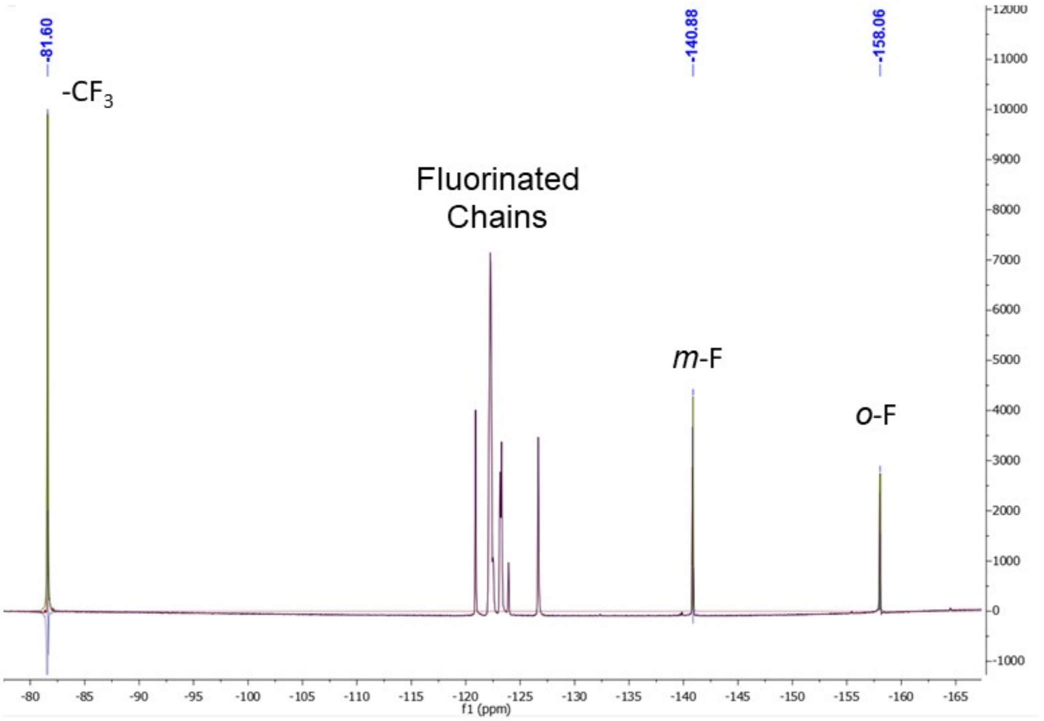

Fig. S3: ^{19}F-NMR of compound **2**.

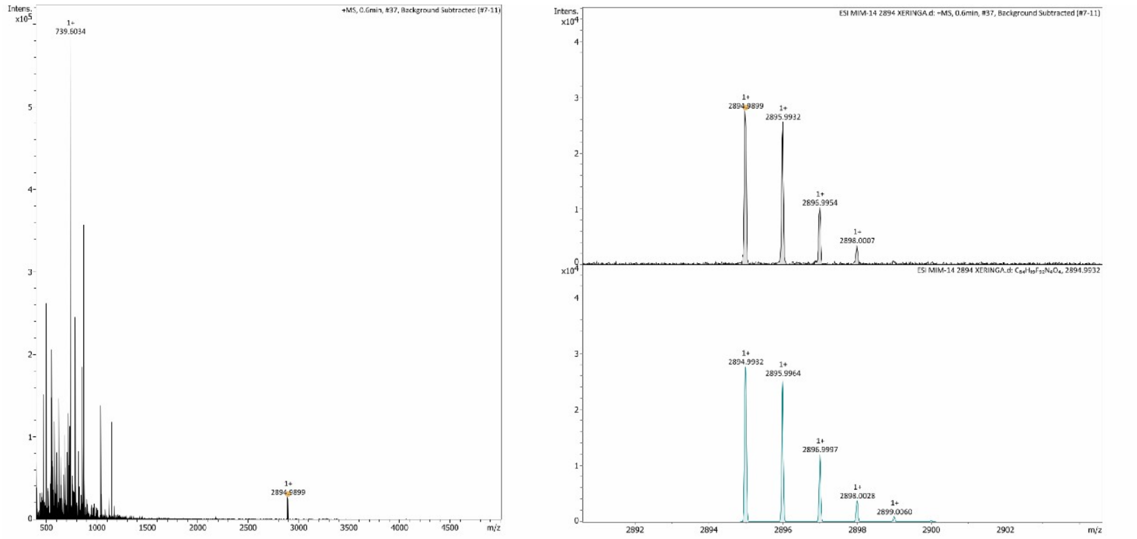

Fig. S4. ESI-FIA-TOF mass spectrometry of compound **2**.

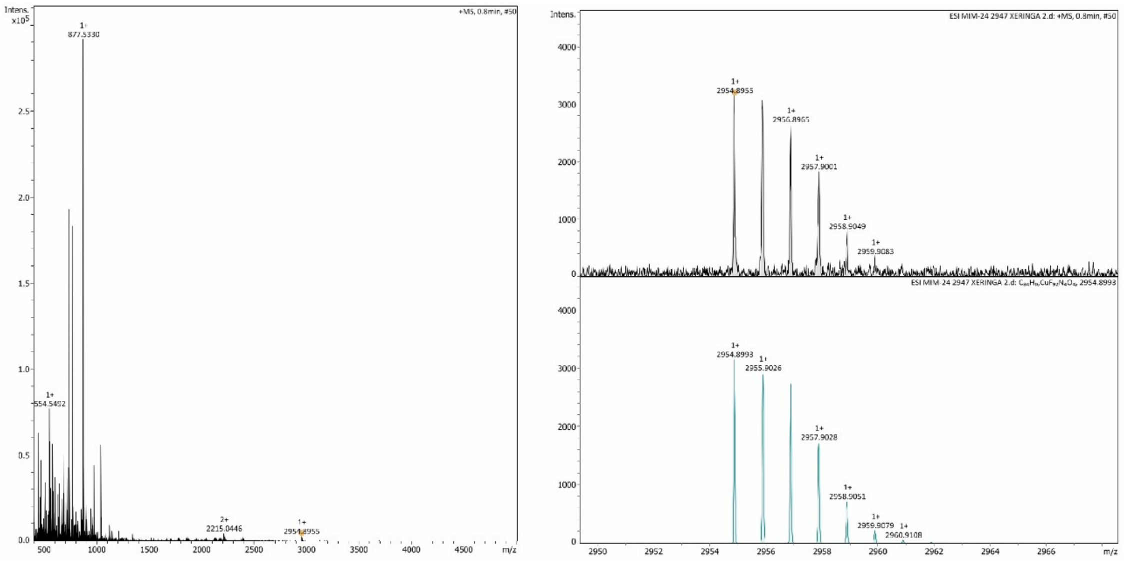

Fig. S5. ESI-FIA-TOF mass spectrometry of compound **3**.

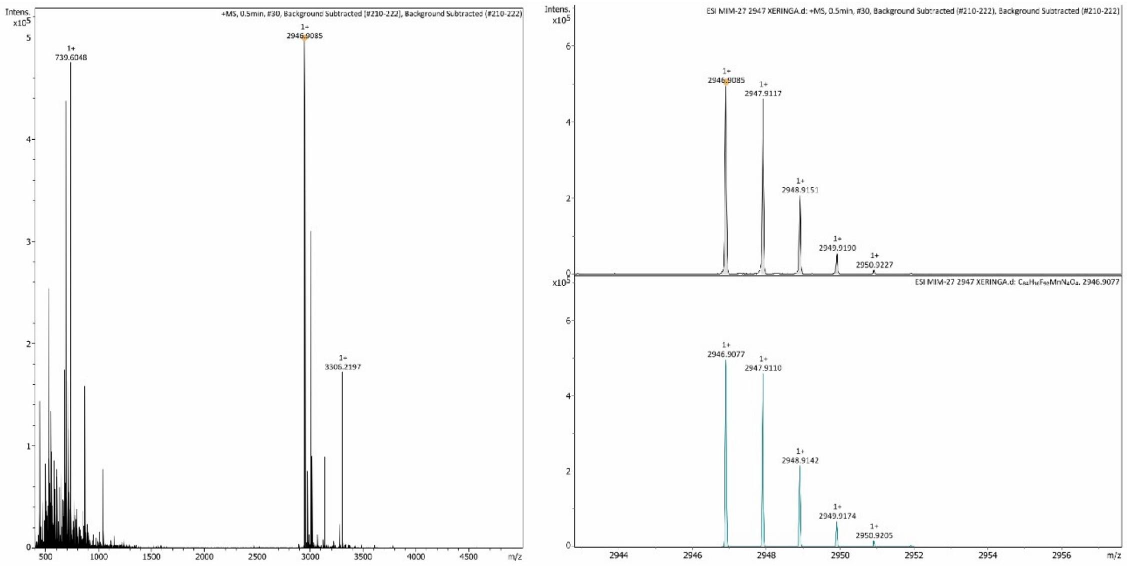

Fig. S6. ESI-FIA-TOF mass spectrometry of compound **4**.

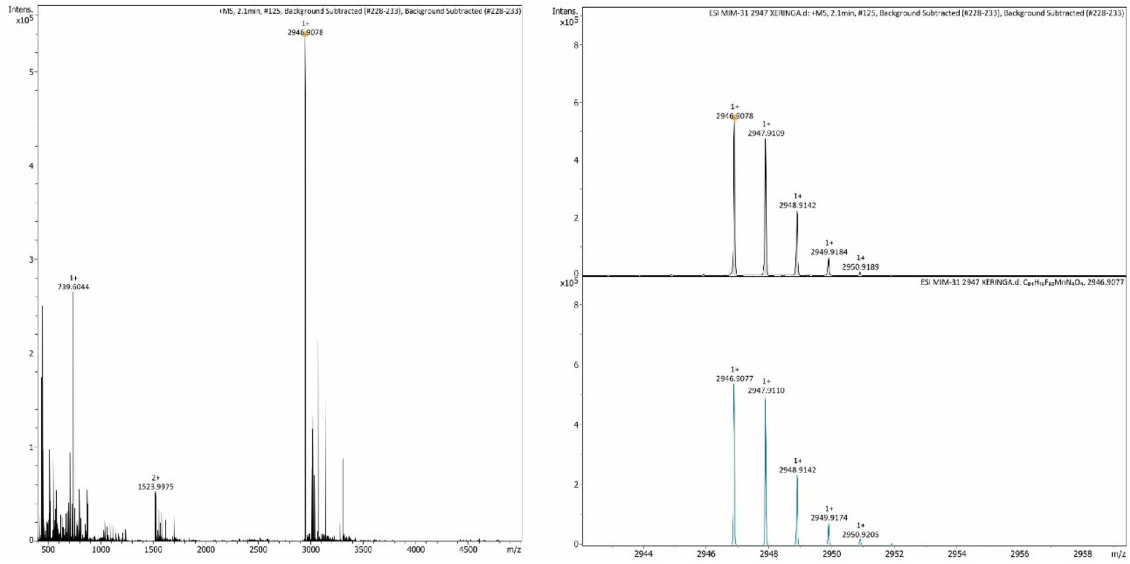

Fig. S7. ESI-FIA-TOF mass spectrometry of compound **4**.

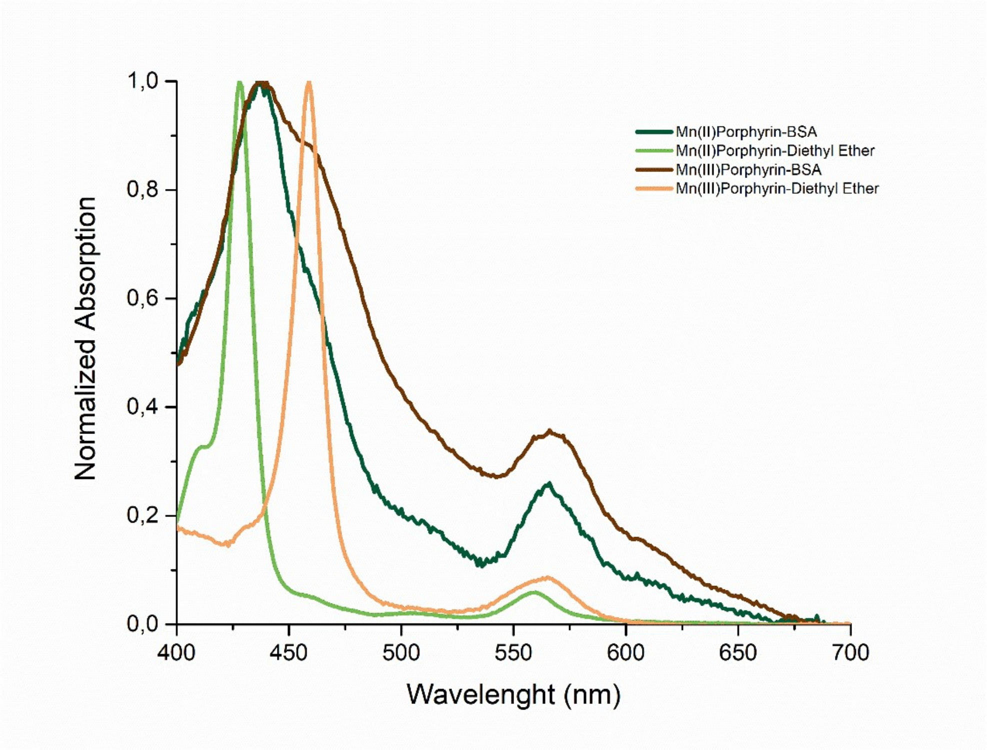

Fig. S8. Absorption spectrum obtained after dissolving **5** is aqueous BSA (brown line), and other absorption spectra revealing that **4** is formed from **5** under these conditions.

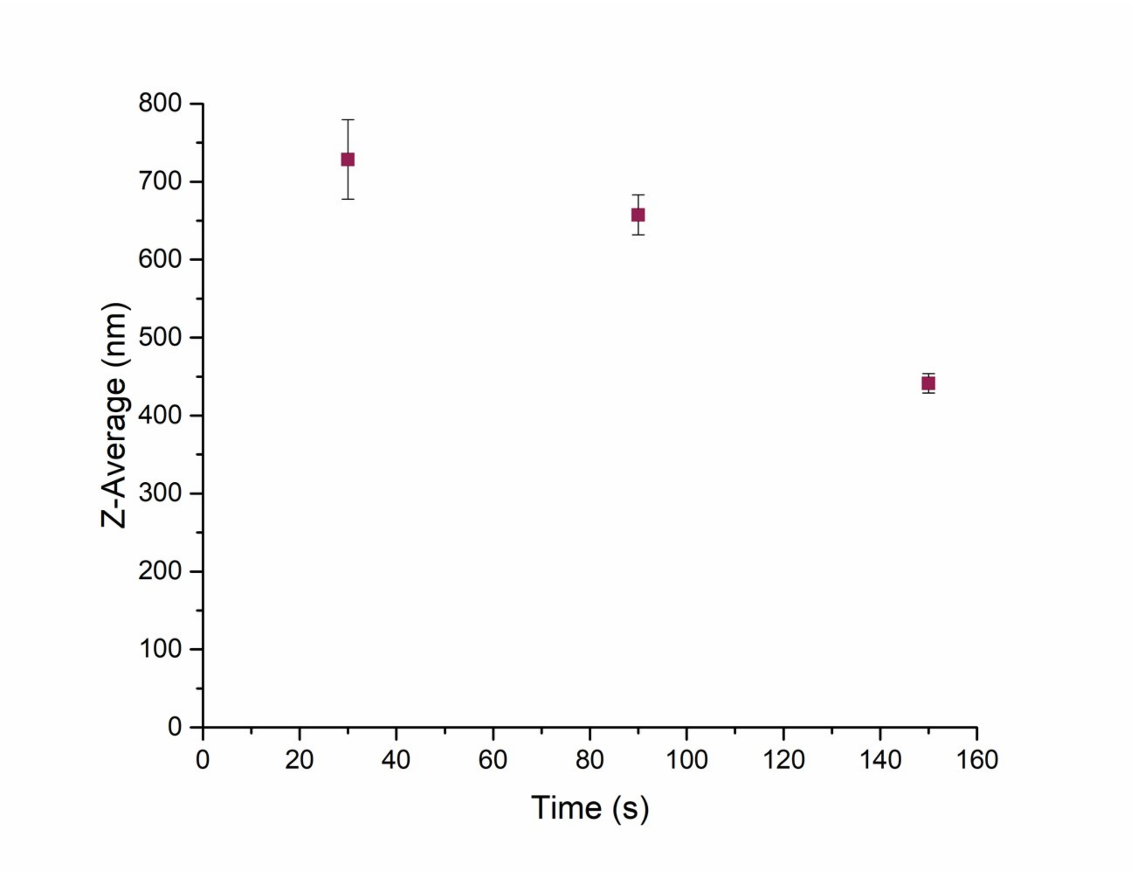

Fig. S9. Sizes of PFP droplets at 25 °C in a 1.7 mL aqueous BSA solution initially in an ice bath and brought 25 °C with 30 sec of sonication (728 nm diameter droplets), with additional &0 sec of sonication at 25 °C (657 nm diameter droplets) and with additional 120 sec of sonication at 25 °C (441 nm diameter droplets).

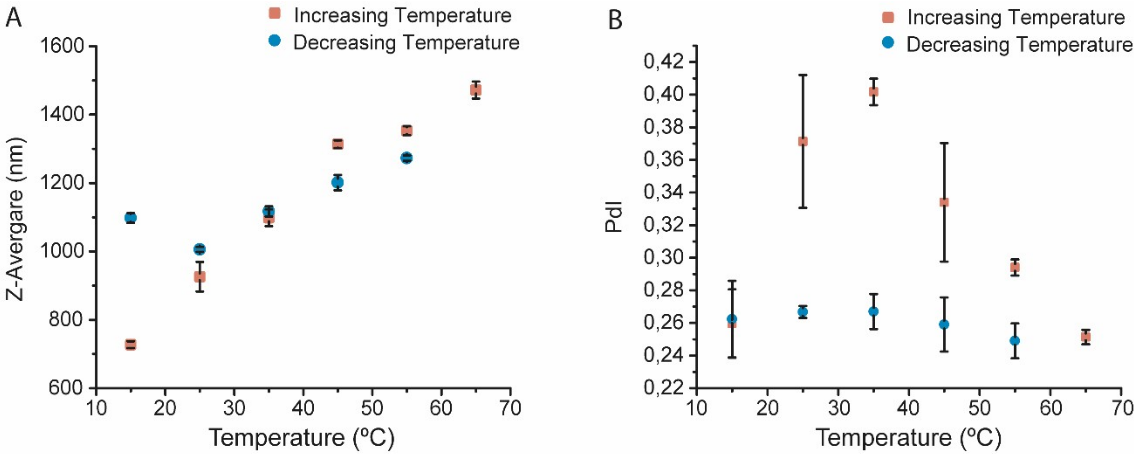

Fig. S10. Temperature dependence of the size of PFP droplets in aqueous BSA loaded with free-base porphyrin **2**. A) Dependence of size on temperature B) Polydispersity indexes (PdI) for each measurement. The Z-Average and PdIs are averages of three consecutive measurements at the same temperature.

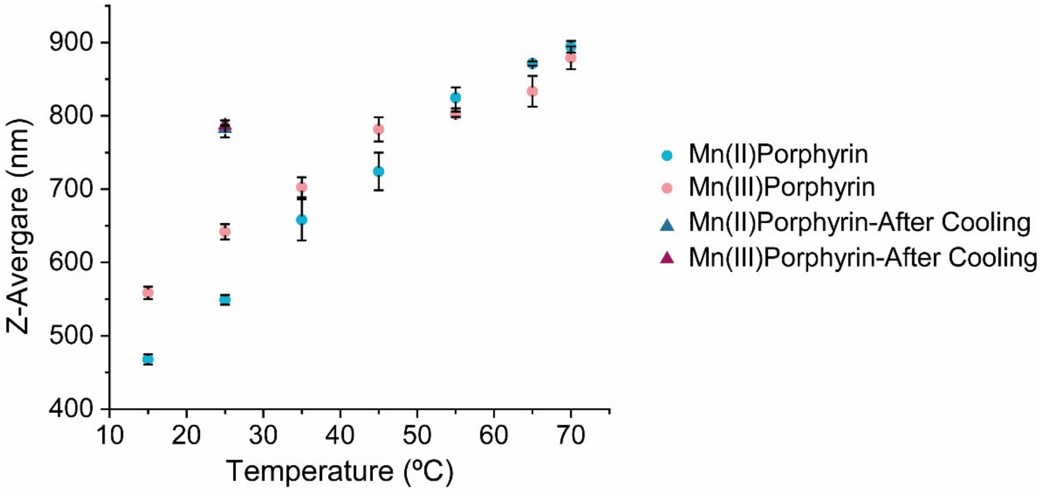

Fig. S11. Temperature dependence of the size of PFP droplets in aqueous BSA loaded with Mn(II)porphyrin **4** and Mn(III)porphyrin **5**. The triangle is the particle diameters after cooling to room temperature.

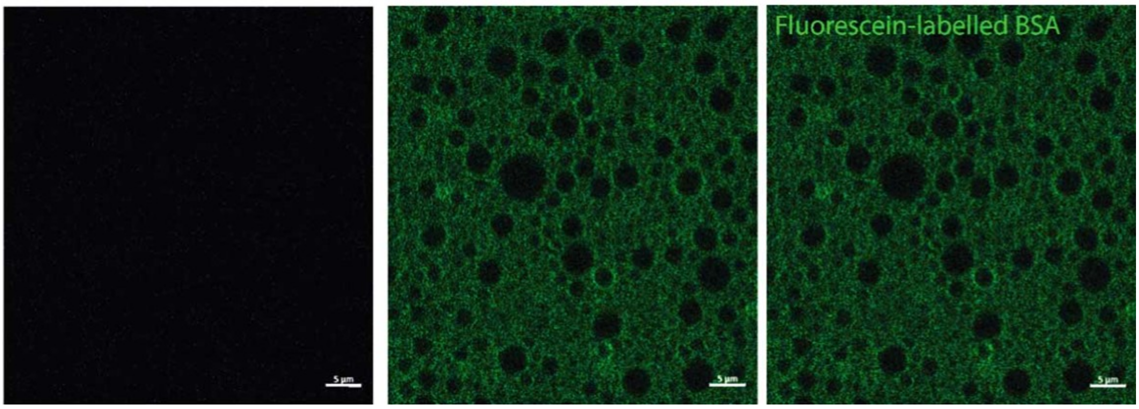

Fig. S12. Confocal microscopy of PFP structures loaded with Mn(II) porphyrin **4** in aqueous fluorescein-labelled BSA (green color). The porphyrin was excited at 405 nm and emission was attempted to be collected at 605/70 but this porphyrin is not fluorescent. Fluorescein-labelled BSA was excited at 488 nm and its fluorescence collected at 525/50 nm. Scale bar is 5 µm.

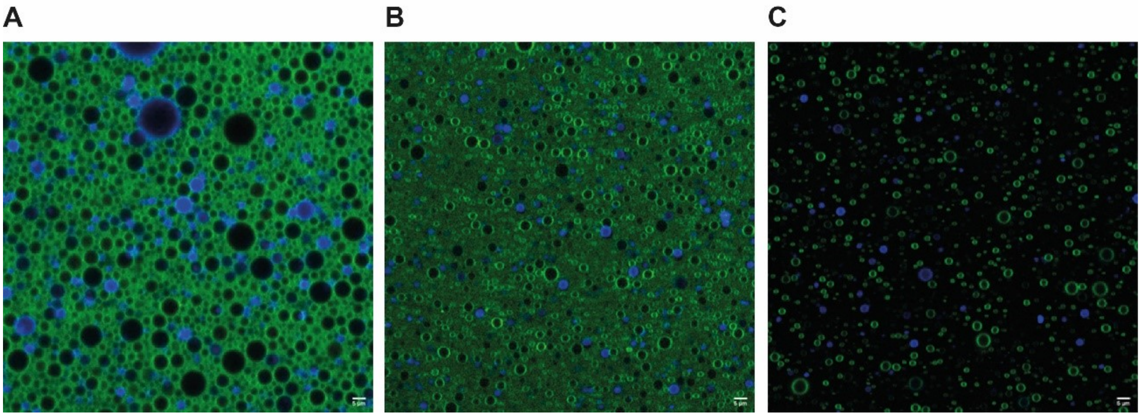

Fig. S13. Confocal microscopy of PFP structures loaded with free-base porphyrin **2** (blue color) in aqueous fluorescein-labelled BSA (green color). These emulsions were prepared from a stock solution of **2** in tetrahydrofuran. B is a 5x dilution of the initial emulsion A with the addition of water, and C is a 20x dilution. The porphyrin was excited at 405 nm and its fluorescence collected at 605/70 nm. Fluorescein-labelled BSA was excited at 488 nm and its fluorescence collected at 525/50 nm. Scale bar is 5 μm.

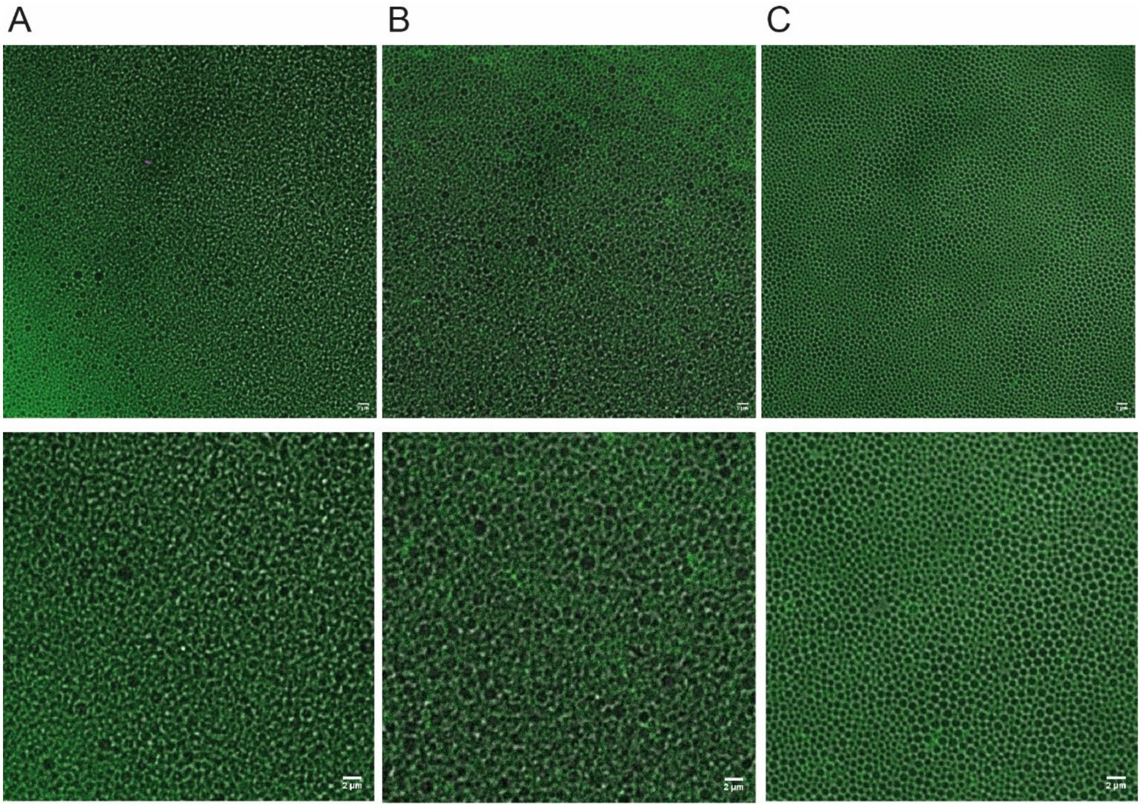

Fig. S14. Confocal microscopy of droplets kept at different temperatures and then allowed to return to room temperature. A) Droplets kept on ice. B) Droplets kept at room temperature. C) Droplets heated to 65 °C for one hour. The lower panels are ampliations of the upper panels.

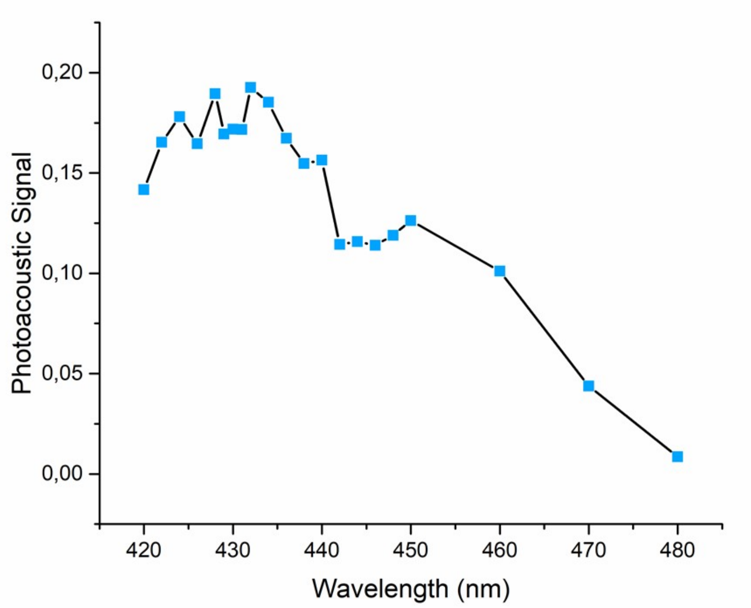

Fig. S15. Photoacoustic spectrum of **4** in PFP droplets dispersed in aqueous BSA. Each data point is an average of the maximum peak of PAWs produced with 50 laser pulses.

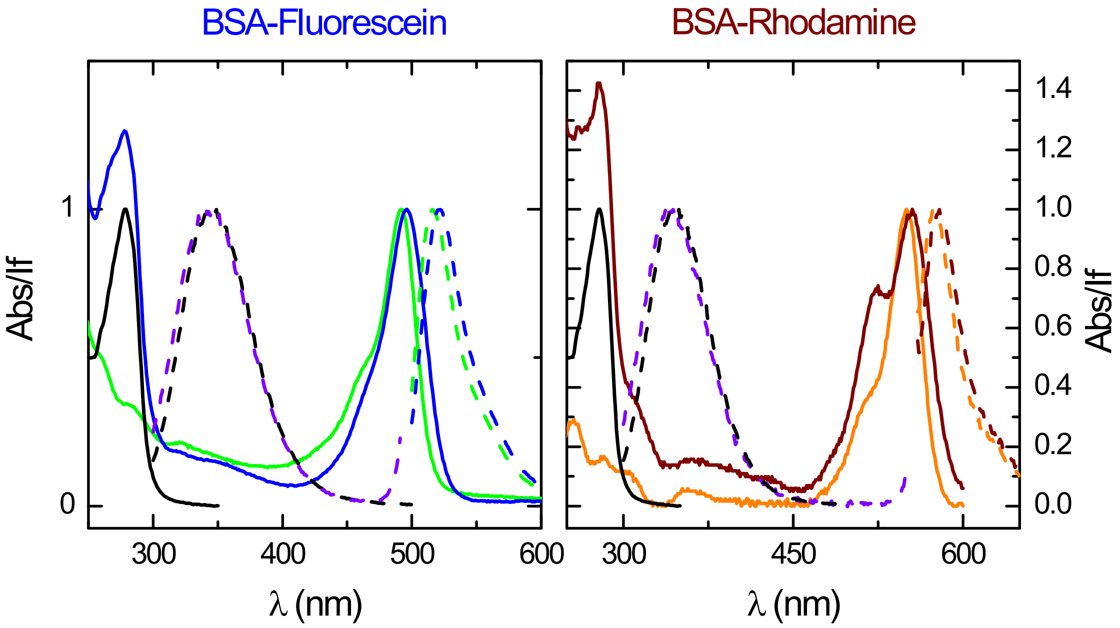

Fig. S16. Absorption (continuous lines) and fluorescence (dashed lines) spectra of BSA conjugates and reactants. Left plot: Fluorescein (— and ---), BSA (— and ---) and BSA conjugate absorption (—) and fluorescence spectra for λ_{exc}=280 nm (---) and for λ_{exc}=492 nm (---). Right plot: tetramethylrhodamine (— and ---), BSA (— and ---) and BSA conjugate absorption (—) and fluorescence spectra for λ_{exc}=280 nm (---) and for λ_{exc}=555 nm (---).
